# Detection of Essential Oil Adulteration Using High-Temperature Gas Chromatography with a Flame Ionization Detector

**DOI:** 10.3390/molecules31132220

**Published:** 2026-06-24

**Authors:** Michal Fulín, Róbert Kubinec, Jaroslav Blaško, Róbert Bodor, Janka Kubincová, Ľubomíra Duhačková, Pavel Farkaš, Radomír Čabala

**Affiliations:** 1 Department of Analytical Chemistry, Faculty of Natural Sciences, Comenius University in Bratislava, Ilkovičova 6, 842 15 Bratislava, Slovakia; fulin1@uniba.sk (M.F.); robert.kubinec@uniba.sk (R.K.); robert.bodor@uniba.sk (R.B.); duhackova2@uniba.sk (Ľ.D.); farkas41@uniba.sk (P.F.); 2 Department of Chemistry and Food Analysis, Food Research Institute, National Agricultural and Food Centre, Priemyselná 4, 824 75 Bratislava, Slovakia; janka.kubincova@nppc.sk; 3 Department of Analytical Chemistry, Faculty of Science, Charles University, Hlavova 8, 128 43 Prague, Czech Republic; cabala@natur.cuni.cz

**Keywords:** essential oils, adulteration, gas chromatography

## Abstract

Essential oils are natural products frequently subject to economically motivated adulteration with cheaper substances like vegetable oils, mineral oils, or organic solvents. This study developed and validated a rapid high-temperature gas chromatography with flame ionization detection (HTGC-FID) method for the simultaneous determination of high-boiling adulterants: triacylglycerides (vegetable oils) and medicinal white oil (mineral oil) in essential oils. The method utilizes on-column injection onto a DB-5 capillary column (30 m × 0.53 mm, 0.88 μm) with a temperature program from 60 to 380 °C and hydrogen carrier gas. Validation parameters demonstrated excellent linearity (R^2^ = 0.9957–0.9978), high repeatability (content RSD < 3%), and sufficient sensitivity (LOQ of 0.03% for triacylglycerides, and 0.63% for medicinal white oil). The method was successfully applied to 20 commercial essential oils. While medicinal white oil was undetected, several samples contained triacylglycerides (up to 3.79%) and other adulterants (up to 52%). Significantly reduced response factors confirmed extensive adulteration in some products. The proposed HTGC-FID method represents a simple, cost-effective, and efficient tool for routine quality control, enabling direct quantification of high-boiling adulterants without tedious sample preparation.

## 1. Introduction

Essential oils are natural, volatile, and complex mixtures of compounds with strong aromas. They are widely used worldwide, and their application continues to grow due to the high demand for pure natural ingredients in many fields. These oily liquids are produced by various aromatic plants as secondary metabolites in their buds, flowers, leaves, stems, twigs, seeds, fruits, roots, wood, or bark, and are stored in secretory cells [1].

The chemical composition, which determines the biological properties of essential oils, is influenced by several factors, particularly the plant species, geographical location, plant part used for harvesting, and method of oil extraction [2,3].

Essential oils are extensively used as food flavorings and for their bactericidal, fungicidal, and insecticidal properties. Recently, they have also found significant applications in human medicine. For example, *Rosmarinus officinalis* (rosemary) and *Mentha piperita* (peppermint) have been shown to have a positive effect on human memory [4].

Interest in essential oils and their compounds is increasing due to their high consumer acceptance [5]. It is estimated that approximately 3000 essential oils are known, of which around 300 have significant commercial importance, especially for the pharmaceutical, food, cosmetic, and fragrance industries [6,7]. Some essential oils are produced on a much smaller scale because of their rarity; however, they are traded at very high prices [e.g., agarwood (€6000–11,000/kg), orris root (€6200–100,000/kg depending on irone concentration), or rose oil (€6000–10,000/kg)]. These prices may be related to the rarity of the raw material, harvesting difficulty, climatic conditions, or low extraction yield. The essential oil market is rapidly expanding and generates several billion dollars annually, which unfortunately also leads to adulteration for dishonest economic profit [8,9].

Various substances are often added to essential oils to increase product volume. This practice, referred to as adulteration, is generally economically motivated. As with other natural commercial products, essential oil producers may consider adulteration relatively easy because some customers do not pay sufficient attention to the quality of the purchased products [10].

Adulteration of essential oils may have harmful effects due to the toxicity of the adulterant or interference with the expected physiological effects of the essential oil. At the same time, it may have a negative economic impact on the essential oil trade [11].

There are certain requirements (price, physicochemical, and sensory properties) that an essential oil adulterant must fulfill. An adulterant is always a lower-value product compared to the pure essential oil. The literature identifies vegetable oils, solvents, other essential oils, and specific chemical compounds as the main adulterants of essential oils [11].

Vegetable oils, for example, are cheaper than essential oils, practically odorless, and have similar density and texture properties. These characteristics make them suitable for the adulteration of essential oils.

Several analytical techniques are used for the detection of essential oil adulteration, including physical, chemical, chromatographic, and spectroscopic methods [12,13,14,15,16,17,18]. The quality of an essential oil may be evaluated using simple techniques such as sensory analysis (organoleptic assessment) or by measuring ester, acid, or carbonyl values, refractive index, density, optical rotation, and melting or boiling points [9].

Advanced separation techniques such as gas chromatography (GC), liquid chromatography (LC), thin-layer chromatography (TLC), or high-performance thin-layer chromatography (HPTLC) may be used to determine the presence of adulterants in essential oils [9]. Gas chromatography coupled with a suitable detector (e.g., MS or FID) may be considered the “gold standard” for essential oil quality control and is highly effective for the identification and quantification of typical volatile constituents. However, significant limitations arise when the adulterants are nonvolatile substances such as vegetable or mineral oils.

Spectroscopic techniques such as FTIR, Raman spectroscopy, and NMR are commonly used for determining vegetable oils as adulterants. FTIR spectroscopy mainly monitors the vibrational band of the carbonyl group, ν(C=O) ≈ 1745 cm^−1^, which is highly intense and practically absent in essential oils. This technique is mainly applied as a screening method with a sensitivity of approximately 3–10% [19,20,21,22].

Raman spectroscopy may be more sensitive than FTIR for the determination of triacylglyceride (TAG) adulterants and enables their detection at approximately the 1–3% concentration level. It mainly monitors the unsaturation of vegetable oils (C=C bonds) and long aliphatic chains. The most significant Raman bands monitored are ν(C=C) ≈ 1655 cm^−1^ and ν(CH_2_) ≈ 1440 cm^−1^.

Very good results are achieved using NMR, where signals of the glycerol backbone (δH ≈ 4.1–4.3 ppm) and olefinic protons (δH ≈ 5.3 ppm) are monitored. The achieved sensitivity is at approximately the 1% level.

The use of UHPLC-MS is also highly suitable, with a limit of quantification (LOQ) of approximately 0.1% achievable while simultaneously identifying the specific vegetable oil. However, both NMR and LC-MS techniques are financially demanding and therefore used only to a limited extent in routine laboratories.

For adulteration with mineral oils, paraffin oil, petroleum jelly oil, and white mineral oil are most commonly used. In the analysis of paraffin oil, paraffins are monitored as markers, which are readily detectable using FTIR, Raman spectroscopy, and NMR. The achieved detection limits are comparable to those obtained for TAG adulterants.

One problem arises in the analysis of petroleum jelly oil and white mineral oil, which mainly consist of branched alkanes and cycloalkanes. If a standard of the mineral oil used for adulteration is not available, quantification using spectroscopic techniques is associated with significant error.

A solution is the use of gas chromatography with an FID, which enables the determination of individual fatty acids in TAG after transesterification or the determination of paraffins in paraffin oil. However, the use of conventional gas chromatography is problematic for the determination of petroleum jelly oil and white mineral oil. The use of high-temperature gas chromatography with FID (HTGC-FID) appears to be a suitable solution because it enables the elution of high-boiling compounds such as TAG.

Unlike spectroscopic methods, the proposed method enables direct separation and quantification of high-boiling adulterants without the need for chemometric data processing. Recently, Kresnik et al. (2026) demonstrated that a unified GC method with a medium-polarity column and temperature program up to 350 °C enables comprehensive chemical profiling of essential oils, supporting the approach of using high-temperature GC for quality control [23].

The aim of this work was to develop a new rapid analytical method for the determination of high-boiling adulterants in essential oils based on gas chromatographic separation with a nonselective FID. The method does not require complex sample preparation or derivatization (except for dilution) and enables the quantification of TAG adulterants as well as mineral oils. At the same time, it allows semiquantitative determination of solvent content and qualitative characterization of the sample composition.

The originality of the proposed method lies in the possibility of analyzing compounds with a very broad boiling point range without discriminatory effects in the injector. These properties are ensured by the appropriate selection of chromatographic and hardware parameters, which allow the analysis of compounds approximately within the C8–C70 range. The method also exhibits a very similar response factor for all analyzed compounds, enabling semiquantitative determination of unknown high-boiling adulterants.

## 2. Results and Discussion

The selection of the column, injection technique, and chromatographic conditions was crucial for achieving the established objectives. A relatively high film thickness of 0.88 µm was chosen due to the need to separate individual components of essential oils and potential solvent adulterants. A final temperature of 380 °C, together with a high carrier gas flow rate of 15.4 mL/min, was required for the elution of TAGs. Hydrogen was selected as the carrier gas to minimize loss of column efficiency at such high flow velocities.

The internal column diameter of 0.53 mm was chosen because of the use of on-column injection. This injection technique was necessary to avoid compound discrimination that would occur in a split/splitless injector due to the extremely wide range of boiling points of the individual components. The initial temperature of 60 °C, together with the use of hexane as the solvent, ensured the formation of a solvent focusing effect at the beginning of the column, which positively influenced separation. Under these optimized conditions, it was possible to use the internal standard C40 for the quantification of TAG adulterants and medicinal white oil, as well as for determining the presence of other substances identified based on the decrease in the response factor of the essential oil.

Table 1 summarizes the basic validation parameters of the analytical method—limit of detection (LOD), limit of quantification (LOQ), calibration curve slope, intercept, relative standard deviation of determined adulterant content at 5% of nominal content from repeated measurements, and linear range. These parameters provide information on the sensitivity, precision, and linearity of the proposed method for the determination of selected adulterants in essential oils.

The results presented in Table 1 indicate that the proposed method exhibits very good linearity throughout the investigated concentration range. The coefficients of determination (R^2^ = 0.9957–0.9978) confirm a high agreement between experimentally measured data and the regression model, demonstrating the suitability of the method for quantitative determination of both types of adulterants over a wide concentration range.

An important parameter is also the detection capability of the method, expressed by the limit of detection and the limit of quantification. For triacylglycerides, a limit of quantification of 0.03% was achieved, indicating very high sensitivity of the method for this group of compounds. In the case of medicinal white oil, the LOQ value is higher (0.63%), which is related to the broader chromatographic profile of this group of compounds. Nevertheless, the achieved detection limit is sufficient for practical applications in the authenticity and quality control of essential oils.

The relative standard deviation (RSD) values of the adulterant content determination were lower than 3%, confirming good repeatability and precision of the method. The low RSD values indicate the stability of the chromatographic system and the suitability of the selected sample injection technique. Repeatability was evaluated using model mixtures containing 5% adulterant in orange oil, and the results demonstrated minimal differences among individual parallel measurements.

For quantitative evaluation, the C40 alkane was used as the internal standard. Its use was essential to minimize the influence of injection volume variability and simultaneously enabled more accurate calculation of response factors for individual essential oil components. This approach increased the reliability of quantitative determination, especially in the analysis of complex mixtures containing compounds with significantly different physicochemical properties.

The achieved validation parameters confirm that the proposed HTGC-FID method is suitable for the detection and quantification of adulterants in essential oils. The combination of linearity, repeatability, and low limits of quantification enables the application of the method in routine monitoring of the quality and authenticity of commercially available essential oils.

**Figure 1:**
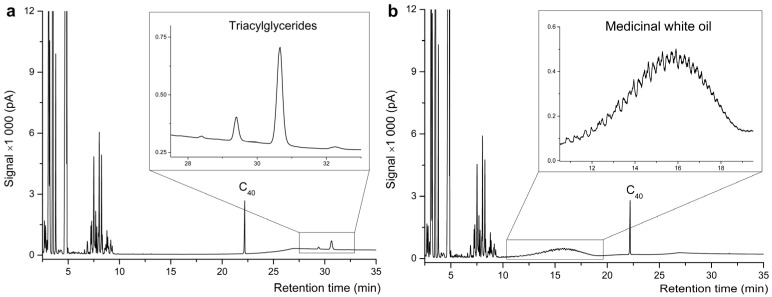
Characteristic chromatographic profile of orange oil with the addition of 2.5% triacylglycerides (**a**), and 5% medicinal white oil (**b**).

Figure 1a,b shows characteristic chromatographic profiles of adulterants in orange oil samples. Figure 1a presents the chromatographic profile of orange oil with the addition of 2.5% TAG from rapeseed oil, while Figure 1b shows the chromatographic profile of orange oil containing 5% medicinal white oil.

The chromatographic records shown in Figure 1a,b demonstrate that the proposed GC-FID method enables effective separation of essential oil components from the investigated adulterants. Sufficient chromatographic resolution of the individual groups of compounds significantly reduces the risk of false positive results in the identification of essential oil adulteration.

For the quantification of TAGs, the sum of all detected acylglycerols was used. This approach was selected because the relative proportion of individual TAGs may differ significantly depending on the type of vegetable oil used as the adulterant. Individual vegetable oils naturally differ in their fatty acid composition, which is subsequently reflected in different chromatographic profiles of TAGs. Therefore, the cumulative approach enables a more universal application of the method without the need to identify each individual TAG.

The chromatographic profile of medicinal white oil clearly shows that it consists of a very broad and complex group of hydrocarbon compounds (the mineral oil hump MOAH) eluting over a wide retention interval (10.5–19.5 min). This property leads to the formation of a broad chromatographic band consisting of many overlapping peaks of hydrocarbon compounds, which necessitates the use of a cumulative approach for the evaluation of mineral oil content in essential oils. The broad band of hydrocarbon compounds negatively affects the sensitivity and limit of quantification of the method. Compared to triacylglycerides, medicinal white oil therefore exhibited higher LOD and LOQ values. Nevertheless, the achieved method parameters remain sufficient for practical application in the authenticity and quality control of essential oils.

The results also confirm that the proposed method is suitable not only for the detection of intentionally added adulterants but also for the indicative assessment of the purity of analyzed samples. Characteristic chromatographic profiles of individual adulterant types may also be used for the identification of the type of added impurity, which is important for quality control of commercially available essential oils and for revealing economically motivated product adulteration.

The results presented in Table 2 indicate that none of the analyzed samples contained medicinal white oil as an adulterant. This finding confirms that this type of adulteration was not present in the analyzed set of samples, or that its concentration was below the limit of quantification of the applied method.

On the other hand, adulteration with vegetable oils, expressed as TAG content, was identified in several samples. Increased TAG content was observed, particularly in samples of *Levisticum officinale* and *Citrus reticulata*, with the highest concentration determined in the lovage oil B sample (3.79%).

An important parameter in the evaluation of oil authenticity was also the relative response factor determined using the FID (see Experimental). The obtained results indicate that authentic essential oils generally exhibit response factors within the range of 0.9 to 1.0. Values close to one correspond to the typical composition of terpene hydrocarbons, which provide a stable and reproducible response on the FID.

A slight decrease in the relative response factor (RRF) may be caused by the natural presence of oxygenated compounds (alcohols, aldehydes, ketones, or esters), which exhibit a lower response on the FID compared to pure hydrocarbons. However, this decrease usually does not exceed 10%. Therefore, response factor values lower than 0.9 indicate the presence of foreign substances, which in most cases are organic solvents used for diluting essential oils. The predominance of terpene hydrocarbons in authentic essential oils results in average response factors close to unity. Prediction models developed for GC-FID demonstrated that hydrocarbons exhibit response factors very close to one, whereas oxygenated compounds provide lower responses. Consequently, the natural occurrence of oxygenated constituents generally causes only a moderate decrease in the average response factor, while substantially lower values are associated with the presence of foreign compounds such as solvents or polyols. Absolute quantification studies of conifer essential oils further confirmed that terpene-rich essential oils exhibit average response factors close to unity [24,25,26].

The most significant deviations were observed in samples of peppermint (*Mentha piperita*), lemon (*Citrus limon*), and lovage oil B (*Levisticum officinale*), where very low response factors (0.57, 0.65, and 0.33, respectively) together with high solvent contents were recorded. These results clearly indicate significant dilution of the analyzed samples and confirm the suitability of the proposed method for detecting essential oil adulteration.

The results also demonstrate that the combination of RRF determination and direct analysis of adulterants represents an effective approach for evaluating essential oil adulteration. The method enables not only the identification of specific types of adulterants but also an indicative assessment of the degree of adulteration of the analyzed samples.

**Figure 2:**
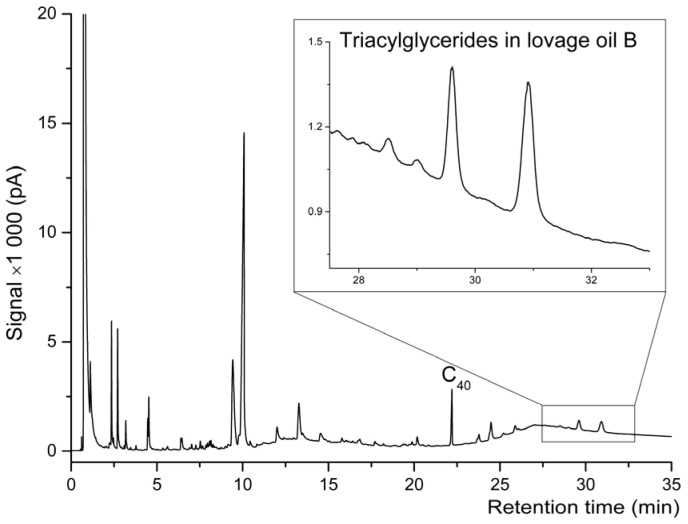
Chromatographic profile of the lovage oil B sample.

Figure 2 shows the chromatographic record of the lovage oil B (*Levisticum officinale*) sample, which represented one of the most heavily adulterated analyzed samples.

From the chromatographic profile shown in Figure 2, the presence of TAG can be clearly identified. The characteristic broad peaks eluting at high retention times correspond to the vegetable oil used as the adulterant. Based on quantitative evaluation, the TAG content in this sample was determined to be 3.79%, which was the highest measured value among all analyzed samples.

In addition to TAGs, the presence of other adulterants could also be identified from the chromatographic record and the reduced RRF value. The RRF of the sample reached a value of 0.33, representing a significant decrease compared to values typical for authentic essential oils. Such a low value cannot be explained solely by the natural presence of oxygenated compounds in the oil—it indicates substantial dilution of the sample with substances exhibiting a low response on the FID.

Based on the evaluation of the chromatographic profile, the content of other adulterants in the sample was estimated to be approximately 52%. This represents an extremely high degree of adulteration that significantly affects both the quality and authenticity of the analyzed product. Such an amount of added solvents may substantially alter the physicochemical properties of the essential oil, its aroma profile, biological activity, and overall product value.

The results of the lovage oil B sample analysis also demonstrate the high selectivity and practical applicability of the proposed GC-FID method. The method enables simultaneous detection of multiple types of adulterants in a single analysis while providing not only qualitative information about the presence of foreign substances but also their approximate quantification. The combination of chromatographic profiling, TAG determination, and RRF evaluation, therefore, appears to be an effective tool for the authenticity and quality control of commercially available essential oils.

## 3. Materials and Methods

### 3.1. Samples and Chemicals

Essential oil samples were purchased from local suppliers and distributors operating on the Slovak market. Commercially available essential oils declared as 100% natural products were selected for analysis. Certified reference materials for essential oils are not commercially available. Therefore, the orange essential oil used for calibration was independently verified as authentic by GC-MS analysis. After purchase, the samples were stored in sealed dark glass vials at 20–25 °C without direct light exposure and analyzed within three months to minimize possible changes in composition caused by oxidation or evaporation.

Rapeseed oil and white petroleum jelly were used as model adulterants and were purchased from a local retail network. Rapeseed oil was selected as a representative TAG adulterant due to its low cost, availability, and physicochemical properties similar to those of essential oils. White petroleum jelly was used as a model mineral oil representing high-boiling hydrocarbon adulterants.

Chromatographic-grade n-hexane (99%) was used as the dilution solvent for sample and calibration solution preparation. Tetracontane (C40) was used as the internal standard. C40 is a non-naturally occurring compound whose retention time falls beyond the elution window of essential oils and the targeted adulterants. Both chemicals were obtained from Sigma-Aldrich (St. Louis, MO, USA).

### 3.2. Preparation of Solutions

The internal standard, n-alkane C40, was prepared in n-hexane at a concentration of 500 mg/L. All essential oil solutions were prepared for analysis by dissolving 100 mg of essential oil, added with a 200 µL precision microsyringe for density determination, in 1 mL of this C40 solution (without further dilution). The density of the analyzed essential oils was in the range of 0.84–0.97 g/mL. Thus, the final concentration of the internal standard in the analyzed solution is in the range 461–456 mg/L.

Model adulterated samples were prepared by gravimetric mixing of orange essential oil with defined amounts in the range of 0.02–20% (*w*/*w*) of vegetable (rapeseed) or mineral (medicinal white) oil. The prepared mixtures were thoroughly homogenized and subsequently prepared for analysis in the same manner as pure essential oil samples.

### 3.3. GC Analysis

The separation of essential oil samples was carried out using a gas chromatography system consisting of an Agilent 6890N gas chromatograph equipped with a flame ionization detector (FID) (Agilent Technologies, Santa Clara, CA, USA). The FID temperature was set to 380 °C, with an air flow rate of 400 mL/min and a hydrogen flow rate of 35 mL/min.

Separation was performed on a DB-5 capillary column (30 m × 0.53 mm I.D., film thickness 0.88 μm; Agilent Technologies) containing a 5% phenyl-methylpolysiloxane stationary phase.

Chromatographic conditions included direct injection of 1 µL of sample using an autosampler in cool on-column (COC) injection mode. Hydrogen was used as the carrier gas at a constant flow rate of 15.4 mL/min. The injector temperature was programmed in oven-track mode with an offset of +3 °C to minimize discrimination effects during the injection of high-boiling compounds.

The chromatographic oven temperature program started at 60 °C. Subsequently, the temperature was increased at a rate of 12 °C/min to 380 °C, where it was held for 8 min.

The peak area of the essential oil is expressed as the sum of all peaks eluting just after the solvent (hexane) up to 10.5 min. The upper integration limit of 10.5 min was selected experimentally. Under the optimized chromatographic conditions, all volatile constituents of the analyzed essential oils eluted (terpenes, terpenoids, and aromatic compounds) before this time, whereas the broad chromatographic zone corresponding to medicinal white oil started at approximately 10.5 min. The peak area of medicinal white oil is expressed as the area of a single peak (mineral oil hump, MOAH) eluting between 10.5 and 19.5 min. The retention time of C40 is 22.2 min. The TAG peak area is expressed as the sum of peaks eluting between 27.5 and 33 min.

The content of other adulterants was calculated from the decrease in the relative response factor (RRF) of the essential oil, which was calculated using the formula:

$$RRF=\frac{A_{x}/c_{x}}{A_{IS}/c_{IS}}$$

where

- *A_x_* = area of all peaks in the essential oil components
- *c_x_* = concentration of the essential oil
- *A_IS_* = peak area of the internal standard C40
- *c_IS_* = concentration of the internal standard C40

An RRF value below 0.9 already indicates the presence of other adulterants, such as solvents, polyethylene glycol, etc.

### 3.4. Method Validation

Limits of detection (LOD) and quantification (LOQ) were determined according to IUPAC recommendations as the lowest analyte concentrations at which the analytical signal could still be distinguished from baseline noise. The following criteria were used for calculation:

- LOD = concentration at which S/N = 3
- LOQ = concentration at which S/N = 10,

where S represents the height of the analytical peak, and N represents the amplitude of the baseline noise. This approach proved to be the most suitable considering the characteristics, shape, and width of chromatographic zones obtained during the elution of adulterants.

Method linearity was evaluated using the coefficient of determination (R^2^) of calibration curves constructed from the dependence of the ratio of adulterant peak areas to the internal standard peak area on the concentration of the monitored adulterants.

Method precision was expressed as the relative standard deviation (RSD) of adulterant content determined from three replicate measurements of a model mixture containing 5% adulterant in orange oil.

Each sample was analyzed in three independent preparations, and each prepared sample was analyzed twice (total 6 measurements per sample). The reported results are expressed as the arithmetic mean.

The semiquantitative determination of solvent content (other adulterants, OA) was based on the decrease in the relative response factor (RRF) according to the following formula:

$$OA\;({\%\;}_{w/v})=(0.9-\frac{{RRF}_{sample}}{{RRF}_{reference}})\times 100$$

where:

*RRF_reference_* is the RRF value for authentic (non-adulterated) essential oils, determined to be 1.0 (based on the measurements of pure samples listed in Table 2 with RRF values of 0.91–1.00). For *RRF_sample_*, values from Table 2 only less than 0.9 are used to calculate OA.

## 4. Conclusions

The aim of this work was to develop and validate a new analytical method based on HTGC-FID for the determination of high-boiling adulterants, specifically vegetable oils (TAGs) and mineral oils (medicinal white oil), in commercially available essential oils. Emphasis was placed on the simplicity of sample preparation (only dilution in a suitable solvent), the ability to separate compounds over a wide boiling point range (approximately C8–C70), and the elimination of discriminatory effects in the injector through on-column injection.

The proposed chromatographic conditions (DB-5 column with a film thickness of 0.88 µm, final temperature of 380 °C, hydrogen flow rate 15.4 mL min^−1^) enabled efficient separation of essential oil components from the investigated adulterants even despite extreme differences in boiling points. Validation parameters confirmed the suitability of the method for quantitative determination: coefficients of determination reached values of R^2^ = 0.9957–0.9978, repeatability (RSD) was lower than 3%, and the limit of quantification for triacylglycerides was 0.03%, while for medicinal white oil it was 0.63%.

Application of the method to 20 commercially available essential oils revealed that none of the samples contained medicinal white oil. On the other hand, adulteration with vegetable oils was identified in several samples (highest TAG content of 3.79% in the lovage oil B sample), together with the presence of other adulterants, where their content reached up to 52% in some cases (lovage oil B). Significantly reduced relative response factors (e.g., 0.33 for lovage oil B, 0.57 for peppermint, and 0.65 for lemon) clearly confirmed adulteration of these products.

The developed COC-HTGC-FID method proved to be a reliable, rapid, and economically accessible tool for routine authenticity control of essential oils. It enables simultaneous detection and determination of vegetable oils, mineral oils, and semiquantitative determination of other adulterants in a single analysis. Compared to spectroscopic methods (FTIR, Raman, NMR), it provides direct separation and quantification without the need for chemometric data processing. The proposed GC-FID method is suitable for routine quality control of commercially available essential oils because it is simple, relatively fast, and more economically accessible than NMR or LC-MS methods, while providing sufficiently sensitive and reproducible results. The originality of the method lies mainly in its ability to analyze compounds with an exceptionally wide boiling point range without discriminatory effects, which makes it particularly suitable for the effective detection of economically motivated adulteration of essential oils in commercial applications.

## Figures and Tables

**Table 1:** Parameters of the analytical method for the determination of triacylglycerides and medicinal white oil: concentration range, calibration curve slope, intercept, limits of detection (LOD), limits of quantification (LOQ), and relative standard deviation (RSD) from repeated measurements.

Type of Adulterant	Concentration Range (%*w*/*v*)	Slope ^1^	Intercept	R^2^	LOD (%*w*/*v*)	LOQ (%*w*/*v*)	RSD ^2^ (%)
Triacylglycerides	0.03–20	0.1031	0.0079	0.9978	0.01	0.03	2.4
Medicinal white oil	0.63–20	0.2633	0.1448	0.9957	0.20	0.63	2.7

^1^ A 10-point concentration scale was used for calibration, enabling accurate determination of the linear range of the method. ^2^ The value was obtained from three parallel measurements of a model mixture containing 5% adulterant in orange oil.

**Table 2:** Relative response factors of individual essential oils together with the content of identified adulterants, expressed as the content of medicinal white oil (MWO), triacylglycerides (TAG), and the presence of other adulterants (OA).

Essential Oil	Botanical Name	RRF	MWO (%*w*/*v*)	TAG (%*w*/*v*)	OA (%*w*/*v*)
Sandalwood	*Santalum album*	0.84	-	0.05	6
Pine tree A	*Pinus sylvestris*	0.94	-	-	-
Pine tree B	*Pinus sylvestris*	1.00	-	-	-
Fir tree	*Abies alba*	1.00	-	-	-
Eucalyptus A	*Eucalyptus globulus*	1.00	-	-	-
Eucalyptus B	*Eucalyptus globulus*	0.85	-	-	5
Rose apple	*Syzygium jambos*	0.99	-	-	-
Honeydew	*Cucumis melo inodorus*	0.77	-	-	13
Rosemary	*Rosmarinus officinalis*	0.96	-	-	-
Mint	*Mentha piperita*	0.56	-	-	34
Cumin	*Cuminum cyminum*	0.98	-	-	-
Tea Tree A	*Melaleuca alternifolia*	0.86	-	-	4
Tea Tree B	*Melaleuca alternifolia*	0.91	-	-	-
Lemon	*Citrus limon*	0.65	-	-	25
Myrtle	*Myrtus communis*	0.94	-	-	-
Siberian Fir	*Abies sibirica*	0.96	-	-	-
Wintergreen	*Gaultheria procumbens*	0.74	-	-	16
Lovage oil A	*Levisticum officinale*	0.73	-	0.08	17
Lovage oil B	*Levisticum officinale*	0.33	-	3.79	52
Tangerine	*Citrus reticulata*	0.95	-	0.68	-

## Data Availability

Data is contained within the article. Further inquiries can be directed to the corresponding author.

## Abbreviations

The following abbreviations are used in this manuscript:

TAG	triacylglycerides
HTGC-FID	High temperature gas chromatography with flame ionization detector
COC	Cool on-column injection
RRF	Relative response factor
